# Association of the Big Five personality traits with cognitive decline: a 24-week multidomain intervention study

**DOI:** 10.3389/fpsyt.2026.1797887

**Published:** 2026-03-19

**Authors:** Jae Youn Jung, Chang Hyung Hong, Yoo Kyoung Park, So Young Moon, Jee Hyang Jeong, Hee Kyung Park, Seong Hye Choi, Joung Hwan Back, Yong Hyuk Cho

**Affiliations:** 1 Department of Psychiatry, Ajou University School of Medicine, Suwon, Republic of Korea; 2 Graduate School of East-West Medical Science, Kyung Hee University, Yong-in, Republic of Korea; 3 Department of Neurology, Ajou University School of Medicine, Suwon, Republic of Korea; 4 Department of Neurology, Ewha Woman’s University Mokdong Hospital, Seoul, Republic of Korea; 5 Department of Neurology, Ewha Woman’s University School of Medicine, Seoul, Republic of Korea; 6 Department of Neurology, Inha University Hospital, Incheon, Republic of Korea; 7 Daejeon University Convergence and Open Sharing System in Biohealth Sciences Project Group, Daejeon, Republic of Korea

**Keywords:** cognitive decline, dementia prevention, multidomain intervention study, personality traits, SUPERBRAIN

## Abstract

**Introduction:**

Multidomain lifestyle interventions have been implemented internationally to improve cognitive function affected by neurodegenerative changes; however, treatment effects vary across individuals. Personality traits are established correlates of cognitive function, but their potential association with individual differences in response to such interventions remains unclear.

**Methods:**

This 24-week prospective study investigated the associations between personality traits and cognitive trajectories among participants within the SoUth Korean study to PrEvent cognitive impaiRment and protect BRAIN health through lifestyle intervention in at-risk older adults (SUPERBRAIN) randomized controlled trial. A total of 136 older adults (mean age = 71.6 years; 64% women) who completed the intervention were included. Personality traits were assessed at baseline using the ten-item Big Five Inventory, and cognitive function was evaluated using the Korean Mini-Mental State Examination at baseline and follow-up. Logistic regression models were adjusted for sociodemographic factors, family history of dementia, health behaviors, depressive symptoms, and daily functioning.

**Results:**

Among the five personality domains, only extraversion was significantly associated with reduced odds of cognitive decline within the intervention group over 24 weeks (fully adjusted model: OR = 0.35; 95% CI: 0.15–0.82).

**Discussion:**

These findings suggest that personality traits may be associated with cognitive stability and highlight the importance of considering personality profiles when understanding inter-individual variability in multidomain dementia prevention strategies.

## 1 Introduction

Cognitive impairment is an increasing global health concern, particularly in aging societies, with major efforts focused on preventing neurodegenerative diseases, including Alzheimer’s disease (1). Recognized modifiable risk factors for dementia include lifestyle-related factors, vascular and metabolic disorders, and psychosocial determinants (2). Several clinical trials have investigated the effects of multidomain lifestyle-based interventions targeting these risk factors to delay cognitive decline and the onset of dementia, such as the Prevention of Dementia by Intensive Vascular Care, the Finnish Geriatric Intervention Study to Prevent Cognitive Impairment and Disability (FINGER), and the World-Wide FINGERS Network (WW-FINGERS) (3–5). Collectively, these studies indicate that multidomain lifestyle interventions are both practical and beneficial for older adults at risk of cognitive decline. However, despite overall group-level benefits, cognitive outcomes following such interventions vary substantially across individuals, limiting their translational impact.

Personality traits— i.e., relatively stable patterns of thoughts, emotions, and behaviors (6)— are plausible factors associated with the heterogeneity in cognitive outcomes after multidomain interventions (7, 8). This influence may operate through multiple pathways, including coping styles with stressful life events, health behaviors, and intelligence levels (9–11). Among the Big Five traits— neuroticism, extraversion, openness, agreeableness, and conscientiousness—higher openness has been linked to better cognitive performance (12, 13), whereas lower neuroticism and higher conscientiousness are associated with reduced cognitive decline (14). However, these studies are primarily observational, and to date, it remains unclear whether personality traits are predictive of individual differences in cognitive changes achieved within multidomain interventions.

Therefore, this study investigated the associations between personality traits and cognitive trajectories among participants following a 24-week multidomain cognitive intervention using data from the “SoUth Korean study to PrEvent cognitive impaiRment and protect BRAIN health” (SUPERBRAIN) randomized controlled trial (15). This study aimed to explore the inter-individual variability in cognitive outcomes within the intervention group and to determine how baseline personality traits relate to these changes. To our knowledge, this is the first prospective study to evaluate the potential associations of personality traits with cognitive outcomes within a 24-week prospective multidomain intervention framework.

## 2 Methods

### 2.1 Study design and participants

This randomized controlled study implemented a 24-week multidomain lifestyle intervention among community-dwelling older adults aged 60–80 years in South Korea who were enrolled in 2019. The intervention was based on the SUPERBRAIN trial design: a multicenter, outcome assessor–blinded study with a three-parallel-arm design (15–17). A per-protocol analysis was performed, including only the 136 participants who completed the 24-week multidomain intervention without any missing follow-up data. Due to the lack of individual-level adherence data, the analysis focused on the association between baseline personality and cognitive outcomes among those who adhered to the protocol. The 24-week multidomain intervention targeted modifiable dementia risk factors and consisted of vascular and metabolic risk management, nutritional guidance, physical exercise, cognitive training, social activity, and motivational enhancement. All participants provided written informed consent.

### 2.2 Assessment of personality traits

Personality traits were assessed at baseline using the Big Five Inventory-10 (18), which includes two items for each of the five dimensions (extraversion, agreeableness, conscientiousness, neuroticism, and openness), rated on a five-point Likert scale (1 = “strongly disagree” to 5 = “strongly agree”). After recoding reverse-keyed items, a separate score was calculated for each of the five dimensions by averaging the two corresponding items for every participant. Consequently, each individual was characterized by a distinct profile consisting of five continuous scores, with higher scores indicating a stronger expression of the trait.

### 2.3 Cognitive outcomes

Cognitive function was assessed at baseline and 24-week follow-up using the Korean version of the Mini-Mental State Examination (19). Cognitive decline was operationally defined as a decrease of 1 point or more in the K-MMSE total score from baseline to 24 weeks. This threshold was informed by prior longitudinal studies identifying an annual decline of ≥1 point on the MMSE as a clinically significant indicator of cognitive impairment in community-dwelling older adults (20, 21). Given the 24-week (6-month) follow-up period of the current study, a 1-point reduction represents a more stringent criterion than the conventional annual threshold, effectively capturing individuals whose cognitive trajectory deviates from normal age-related changes. Participants with stable or improved scores were classified as having no decline.

### 2.4 Covariates

Baseline covariates included sociodemographic characteristics (age, sex, and years of education), family history of dementia (first-degree relative with a clinical diagnosis of dementia), health behaviors (smoking status and alcohol consumption), depressive symptoms, and functional status. Depressive symptoms were assessed using the validated Korean version of the 15-item Geriatric Depression Scale–Short Form (22). Functional status was evaluated with the Korean version of the Bayer Activities of Daily Living scale (23) and the Korean Instrumental Activities of Daily Living scale (24). Education level was categorized as ≤6, 7–9, 10–12, and >12 years.

### 2.5 Statistical analyses

Baseline characteristics were summarized using descriptive statistics. Between-group comparisons (decline vs no decline) were conducted using χ² tests for categorical variables and independent t tests for continuous variables. Given the lack of prior evidence regarding the role of personality traits in the SUPERBRAIN trial, this study was conducted as an exploratory analysis.

The primary analysis examined the association between baseline personality traits and the likelihood of cognitive decline after 24 weeks. We fit binary logistic regression models with the dependent variable coded as decline = 1 (any decrease in K-MMSE from baseline to follow-up) and no decline = 0 (stable or improved K-MMSE). To identify the independent associations between personality traits and cognitive decline, multiple logistic regression analysis was performed by entering all five personality traits simultaneously into the model. This allowed for the evaluation of each trait while controlling for the potential confounding effects of the other four traits.

Three hierarchical models were specified: Model I adjusted for age, sex, education, and baseline K-MMSE; Model II additionally adjusted for family history of dementia and health behaviors (smoking or alcohol consumption); and Model III further adjusted for depressive symptoms (Korean version of the Geriatric Depression Scale-Short Form) and disability measures (Korean version of the Bayer Activities of Daily Living scale and Korean Instrumental Activities of Daily Living scale). The Hosmer–Lemeshow test was used to assess model goodness-of-fit, and multicollinearity among the five personality traits was assessed using the Variance Inflation Factor (VIF). Odds ratios (ORs) with 95% confidence intervals (CIs) were reported. All analyses were conducted using SAS version 9.4 (SAS Institute Inc., Cary, NC), with two-sided P values <.05 considered statistically significant.

### 2.6 Ethical considerations

This study was registered with the Korean Clinical Research Information Service (KCT0005231) and was approved by the Institutional Review Board of Ajou University Hospital (AJIRB-MED-SUR-20-175). All procedures were conducted in accordance with the Declaration of Helsinki and relevant institutional guidelines. Written informed consent was obtained from all participants and their informants.

## 3 Results

### 3.1 Baseline characteristics

Baseline characteristics of the study participants are shown in **Table 1**. Of the 136 participants, 121 (89%) scored ≥24 on the K-MMSE at baseline, whereas 15 (11%) scored <24, indicating cognitive impairment. The mean (SD) age was 71.6 (5.6) years, and 64% of the participants were women. The mean (SD) K-MMSE score was 27.2 (2.4) at baseline and 27.3 (2.4) after the 24-week multidomain intervention. The mean (SD) scores for the Big Five personality dimensions were 2.6 (0.7), 3.3 (0.6), 3.5 (0.8), 2.3 (0.8), and 2.7 (0.9) for extraversion, agreeableness, conscientiousness, neuroticism, and openness, respectively.

**Table 1:** Baseline characteristics of the study participants.

Variable			M(N) ± SD(%)
Cognitive function	K-MMSE (Baseline)		27.2 ± 2.4
	K-MMSE (Baseline)	Normal (score ≥ 24)	121 (89.0)
		Cognitive impairment (score < 24)	15 (11.0)
	K-MMSE (24 weeks follow-up)		27.3 ± 2.4
Big Five Personality Scale	Extraversion		2.6 ± 0.7
	Agreeableness		3.3 ± 0.6
	Conscientiousness		3.5 ± 0.8
	Neuroticism		2.3 ± 0.8
	Openness		2.7 ± 0.9
Socio-demographics	Age		71.6 ± 5.6
	Sex	Men	49 (36.0)
		Women	87 (64.0)
	Education (years)	≤6	42 (30.9)
		7–9	18 (13.2)
		10–12	32 (23.5)
		>12	44 (32.4)
Family history	Family history of dementia	None	108 (79.4)
		Yes	28 (20.6)
Health behaviors	Smoking	Non-smoker	117 (86.0)
		Smoker	19 (14.0)
	Alcohol	Non-drinker	101 (74.3)
		Drinker	35 (25.7)
Disability	Bayer-ADL		1.9 ± 1.1
	K-IADL		0.1 ± 0.2
Depressive symptoms	SGDS-K	Normal (0–7)	108 (79.4)
		Depression (score ≥8)	28 (20.6)

### 3.2 Group comparisons by cognitive change

Participants were divided into two groups based on changes in K-MMSE scores after the 24-week multidomain intervention: the Decline Group (n = 54, 39.7%), defined as those whose scores decreased from baseline, and the No-Decline Group (n = 82, 60.3%), defined as those whose scores remained or improved compared with baseline. As shown in **Table 2**, there were no significant differences in baseline sociodemographic characteristics or Big Five personality trait scores between the ‘cognitive decline’ and ‘no-decline’ groups (all P > 0.05). Specifically, the mean baseline K-MMSE score did not differ significantly between the groups (27.1 vs. 27.4; P = 0.47). Similarly, no significant between-group differences were observed for any of the Big Five traits, family history of dementia, health behaviors, functional status, or depressive symptoms, indicating that the groups were well-balanced at the starting point.

**Table 2:** Baseline characteristics by cognitive change status after 24-week intervention.

Variable			Changes in cognitive function			
			No decline (n = 82)	Decline (n = 54)	χ^2^/t	P
Cognitive function	K-MMSE (Baseline)		82 (60.3)	54 (39.7)	-0.72	0.4710
			27.1 ± 2.3	27.4 ± 2.7		
Big Five Personality Traits	Extraversion		2.5 ± 0.7	2.7 ± 0.8	-1.44	0.1542
	Agreeableness		3.3 ± 0.6	3.3 ± 0.7	-0.38	0.7084
	Conscientiousness		3.5 ± 0.8	3.5 ± 0.8	-0.25	0.7997
	Neuroticism		2.3 ± 0.8	2.4 ± 0.9	-0.42	0.6764
	Openness		2.7 ± 0.9	2.6 ± 0.8	0.16	0.8699
Socio-demographics	Age		71.7 ± 5.4	71.4 ± 5.9	0.28	0.7794
	Sex	Men	32 (39.0)	17 (31.5)	0.80	0.3700
		Women	50 (61.0)	37 (68.5)		
	Education level (years)	≤6	27 (32.9)	15 (27.8)	5.09	0.1656
		7–9	7 (8.5)	11 (20.4)		
		10–12	18 (22.0)	14 (25.9)		
		>12	30 (36.6)	14 (25.9)		
Family history	Family history of dementia	None	65 (79.3)	43 (79.6)	0.005	0.9593
		Yes	17 (20.7)	11 (20.4)		
Health behaviors	Smoking	Non-smoker	69 (84.2)	48 (88.9)	0.61	0.4351
		Smoker	13 (15.9)	6 (11.1)		
	Alcohol	Non-drinker	58 (70.7)	43 (79.6)	1.35	0.2455
		Drinker	24 (29.3)	11 (20.4)		
Disability	Bayer-ADL		1.8 ± 0.9	2.0 ± 1.3	-1.14	0.2565
	K-IADL		0.1 ± 0.2	0.2 ± 0.2	-1.68	0.0944
Depressive symptoms	SGDS-K	Normal (0–7)	63 (76.8)	45 (83.3)	0.84	0.3587
		Depression (Score ≥8)	19 (23.2)	9 (16.7)		

### 3.3 Associations of personality traits with cognitive decline

**Table 3** presents the results of the multiple logistic regression analysis, where all five personality traits were entered simultaneously into the model to identify their independent associations with cognitive decline. In Model I, which adjusted for age, sex, education, and baseline K-MMSE scores, higher extraversion was associated with lower odds of cognitive decline (OR: 0.38; 95% CI: 0.17–0.84; P = 0.02). This association remained essentially unchanged after further adjustment for family history of dementia and health behaviors in Model II (OR: 0.38; 95% CI: 0.19–0.85; P = 0.02). In the fully adjusted Model III, which additionally accounted for depressive symptoms and disability, higher extraversion continued to show a significant association with reduced odds of cognitive decline (OR: 0.35; 95% CI: 0.15–0.82; P = 0.02). This indicates that for every one-point increase in the extraversion score, the likelihood of cognitive decline was reduced by approximately 65%.

**Table 3:** Multiple logistic regression models of the association between baseline personality traits and cognitive decline after a 24-week intervention.

Variable			Model I		Model II		Model III	
			OR (95% CI)	P	OR (95% CI)	P	OR (95% CI)	P
Big Five Personality Traits	Extraversion		0.38 (0.19–0.85)	0.0165	0.38 (0.19–0.85)	0.0185	0.35 (0.15–0.82)	0.0156
	Agreeableness		0.8 (0.36–1.77)	0.5800	0.76 (0.33–1.75)	0.5158	0.92 (0.38–2.25)	0.8538
	Conscientiousness		0.91 (0.49–1.67)	0.7526	0.92 (0.49–1.73)	0.7914	0.84 (0.42–1.66)	0.6127
	Neuroticism		0.69 (0.38–1.25)	0.2222	0.68 (0.37–1.25)	0.2138	0.68 (0.35–1.33)	0.2599
	Openness		1.39 (0.77–2.52)	0.2784	1.31 (0.71–2.41)	0.3861	1.35 (0.71–2.55)	0.3599
Cognitive function	K–MMSE (Baseline)	Normal (score ≥24)						
		Cognitive decline (score < 24)	1.54 (0.08–29.56)	0.7737	1.54 (0.08–30.18)	0.7765	1.54 (0.07–35.61)	0.7878
Socio–demographics	Age		0.94 (0.84–1.05)	0.2589	0.95 (0.85–1.07)	0.3941	0.96 (0.85–1.08)	0.4462
	Sex	Men						
		Women						
	Education level (years)	≤6						
		7–9	0.13 (0.03–0.57)	0.0066	0.12 (0.03–0.56)	0.0070	0.1 (0.02–0.5)	0.0054
		10–12	0.29 (0.08–1.1)	0.0684	0.27 (0.07–1.04)	0.0573	0.29 (0.08–1.28)	0.1025
		>12	0.4 (0.1–1.68)	0.2102	0.38 (0.09–1.65)	0.1981	0.36 (0.08–1.71)	0.1990
Family history	Family history of Dementia	None						
		Yes			1.39 (0.43–4.52)	0.5853	1.39 (0.41–4.67)	0.5987
Health behaviors	Smoking	Non–smoker						
		Smoker			0.95 (0.16–5.58)	0.9564	0.58 (0.09–3.75)	0.5671
	Alcohol	Non–drinker						
		Drinker			2.42 (0.51–11.48)	0.2679	1.39 (0.27–7.09)	0.6895
Disability	Bayer–ADL						0.69 (0.35–1.34)	0.2678
	K–IADL						0.4 (0.01–20.32)	0.6459
Depressive symptoms	SGDS–K	Normal (0–7)						
		Depression (score ≥ 8)					1.94 (0.46-8.12)	0.3648

None of the other Big Five personality traits (i.e., agreeableness, conscientiousness, neuroticism, or openness) was significantly associated with cognitive outcomes in any of the models. All five personality traits were included in the same regression model to adjust for each other’s effects, and no significant multicollinearity was observed (all Variance Inflation Factors < 2.0). The confidence interval for baseline cognitive status was wide (OR: 1.54; 95% CI 0.07–35.61), likely reflecting sparse outcome events and limited precision within this sample. Therefore, this specific coefficient should be interpreted with caution.

## 4 Discussion

Multidomain lifestyle interventions are being increasingly recognized as promising strategies for preventing cognitive decline in aging populations. However, cognitive outcomes following such programs are heterogeneous, as some individuals show marked benefits while others experience little to no cognitive improvement. This study examined whether personality traits were associated with inter-individual variability in cognitive trajectories within the intervention group over 24 weeks, and this association remained significant after adjusting for sociodemographic, clinical, and psychosocial factors. Using hierarchical logistic regression with stepwise adjustment for sociodemographic variables, health behaviors, and baseline clinical factors, we evaluated the independent association between personality traits and cognitive outcomes. Interestingly, although extraversion did not show a significant difference between groups in the initial baseline comparison (P > 0.05), it emerged as a significant factor associated with cognitive stability in the adjusted regression models (Model III, P = 0.0156). This pattern may reflect the underlying covariate structure at baseline; after accounting for these factors, the independent association of extraversion became statistically detectable. Accordingly, our findings should be interpreted as adjusted associations rather than evidence of intervention-specific moderation.

The association between extraversion and cognitive stability is noteworthy given our operational definition of cognitive decline (a ≥1-point drop within 24 weeks). From a clinical perspective, a decrease of 1 point per year in the MMSE score is often considered a representative marker of age-related cognitive decline in community-dwelling older adults (20, 21). Because our follow-up interval was 6 months, a 1-point decline over 24 weeks corresponds to a proportionally faster rate than the commonly used annual threshold. This criterion was intended to reduce the likelihood that the decline classification reflects only trivial score fluctuations over a short interval, although measurement variability cannot be fully excluded. The magnitude of the association is further highlighted by the reported odds ratio (OR = 0.35), indicating that higher extraversion scores were associated with substantially lower odds of being classified in the cognitive decline group. Nevertheless, this estimate should be interpreted cautiously given the short follow-up period and the limited number of decline events.

Previous epidemiologic studies have reported associations between personality and long-term cognitive trajectories. Higher openness has been linked to better cognitive performance (8, 12, 13), whereas lower neuroticism and higher conscientiousness are associated with slower cognitive decline (14, 25). Biological mechanisms may partly underlie these associations. Positron emission tomography studies in the Baltimore Longitudinal Study of Aging have shown that higher neuroticism is linked to greater cortical amyloid and tau burden, whereas conscientiousness is associated with lower deposition, independent of apolipoprotein E genotype and other covariates (26). Complementing this, plasma biomarker data from the same cohort demonstrated that neuroticism was associated with higher glial fibrillary acidic protein and neurofilament light chain, while conscientiousness and extraversion were related to lower levels of these markers (27). These converging findings suggest that personality traits may capture underlying vulnerability or resilience to neurodegenerative processes. Structural neuroimaging studies also indicate that personality traits correspond to gray- and white-matter features, with extraversion associated with occipital cortical surface area, neuroticism with parietal cortical thickness, and openness with anterior–posterior white matter tract integrity (28). Although these results were derived from young healthy adults, they provide evidence that personality is linked to large-scale brain morphology and connectivity in addition to molecular and cellular markers of pathology. Together, these multimodal findings reinforce the hypothesis that personality contributes to brain reserve and resilience across different levels of analysis, from proteinopathy to structural integrity.

Our findings suggest that within the specific framework of the SUPERBRAIN program, an individual’s personality profile —specifically extraversion —is a clinically relevant factor associated with cognitive stability over 24 weeks. Prior studies examining extraversion in relation to cognitive intervention outcomes have reported mixed results (8, 12, 29), which underscores the need to interpret our findings cautiously and to consider program-specific characteristics when comparing across studies.

The design of the SUPERBRAIN intervention may help explain why extraversion emerged as a significant factor in our study. Among the five domains— i.e., cognitive training, vascular risk factor management, physical exercise, nutrition, and motivational enhancement—the inclusion of motivational and socially engaging components may align particularly well with the social and group-based engagement tendencies often observed in extraverted individuals (30, 31). Extraverts are also more likely to participate in social activities, experience positive affect, and benefit from social support (32–34), which may have contributed to more favorable outcomes within the program. Importantly, as individual adherence data across the different intervention modules were not collected in this study, this interpretation that personality traits influenced the degree of engagement remains hypothesis-generating. Based on existing literature, it is plausible to speculate that extraverted individuals may have engaged more intensively or socially with the intervention modules (35–38). However, without quantitative adherence data, this remains a potential mechanism that requires empirical validation in future research.

Nevertheless, these results highlight the importance of viewing personality as a potentially useful framework for tailoring intervention delivery and support in the context of intervention design. Rather than treating personality as a static determinant, it can be used to anticipate how different individuals may interact with lifestyle modifications and to inform strategies that support engagement.

Notably, most prior investigations linking the Big Five traits to cognitive trajectories have been long-term observational studies where higher neuroticism and lower conscientiousness often relate to depressive symptoms or health behaviors over time (39, 40). In contrast, our study focused on a relatively short-term, 24-week multidomain intervention in which the degree of active engagement may have been more relevant than long-term associations of enduring traits. In this setting, personality-related differences in how participants interacted with the intervention modules may have contributed to the observed associations with cognitive stability.

This study has several limitations. First, the relatively small sample size and restriction to older, highly educated Korean adults may have limited the statistical power and generalizability of the findings. Consequently, further cross-cultural and multi-ethnic studies are required to confirm the universal applicability of the association between personality traits and multidomain intervention outcomes. Second, personality traits were assessed using the brief Big Five Inventory-10, which, although efficient, has lower reliability than longer inventories. Third, cognitive outcomes were based on short-term changes using a global screening measure (K-MMSE), which is less sensitive to subtle or domain-specific changes and may be influenced by measurement variability.

Fourth, a significant limitation of this study is the absence of a control-group comparison in the current analysis. While our study was designed to explore the predictive value of personality traits within the intervention group, the lack of a formal comparison with a control group (e.g., usual care) precludes us from confirming whether the observed associations are specific to the SUPERBRAIN intervention. It remains possible that these findings reflect a general prognostic relationship of personality on cognitive change that might also be present in untreated populations. Therefore, the present results should be interpreted as predictive associations rather than intervention-specific causal moderation.

Lastly, the lack of quantitative data on participant adherence and engagement limits our ability to clarify the underlying behavioral mechanisms. While we observed a significant link between extraversion and cognitive stability, we cannot determine whether this was mediated by more intensive participation in the intervention modules. Future research should incorporate objective measures of adherence to better understand how personality interacts with intervention engagement and to further delineate the role of personality as a potential behavioral factor in multidomain lifestyle programs.

Despite these limitations, to our knowledge, this study is among the first investigations within a randomized controlled trial framework to examine associations between personality traits on short-term cognitive outcomes among participants receiving a structured multidomain cognitive intervention. These findings highlight the potential value of integrating personality assessments into dementia prevention programs and suggest that tailoring interventions to personality traits may enhance their effectiveness. Further research using larger and more diverse cohorts, comprehensive personality and cognitive assessments, and incorporating neuroimaging and biomarker data is warranted to clarify the mechanisms underlying these associations and to develop truly personalized intervention strategies.

## Data Availability

The datasets presented in this article are not readily available because of the sensitive nature of participant information. Data may be made available from the corresponding author upon reasonable request. Requests to access the datasets should be directed to Yong Hyuk Cho, brainist.cho@gmail.com.
